# The Referral System between Primary and Secondary Health Care in Saudi Arabia for Patients with Type 2 Diabetes: A Systematic Review

**DOI:** 10.1155/2017/4183604

**Published:** 2017-05-29

**Authors:** Mohammed Senitan, Ali Hassan Alhaiti, James Gillespie, Badar Faiz Alotaibi, George Binh Lenon

**Affiliations:** ^1^Department of Public Health, Faculty of Health Sciences, Saudi Electronic University, Riyadh, Saudi Arabia; ^2^Menzies Centre for Health Policy, Sydney School of Public Health, University of Sydney, Sydney, NSW, Australia; ^3^Nursing Education Departments, King Fahad Medical City, Riyadh, Saudi Arabia; ^4^School of Health and Biomedical Sciences, RMIT University, Bundoora West Campus, Bundoora, VIC 3083, Australia; ^5^Adult Emergency Department, King Fahad Medical City, Riyadh, Saudi Arabia

## Abstract

**Background:**

In Saudi Arabia, the mortality of diabetes is currently reported at 6%. A well-administered referral system is crucial in aiding the management of this disease.

**Method:**

A single reviewer employed a systematic approach to searching the literature databases with regard to the question: what are the attributes of referral systems in Saudi Arabia for patients with type 2 diabetes (T2D)? The results were analysed in order to provide recommendations to improve the Saudi health system.

**Results:**

Twelve primary studies were identified from a systematic search. Overall, the 12 studies did not clearly mention any of the factors of a good referral system. The referral problems identified by this study included patients' unnecessary requests for referral, unstructured referral letters, and unclear dissemination guidelines for referral.

**Conclusions:**

This research attempted to identify the efficiency of the referral processes that were implemented for patients with T2D. The majority of the included studies were completely silent on the main referral factors for patients. If this review is representative of the referral system in Saudi Arabia, then, in the context of T2D, current referrals are unsafe. Further research on the quality of the referral system, taking into account at least some of the WHO referral guidelines, is required.

## 1. Introduction

One of the challenges to Saudi Arabia's health care system is the change in the burden of diseases from communicable infectious diseases to noncommunicable chronic diseases, such as diabetes and cardiovascular illness [1]. Chronic diseases counted for 71% of all mortalities in Saudi Arabia in 2011, while the mortality rate due to diabetes in Saudi Arabia was reported at 6% in 2011 [1]. According to Al-Daghri et al., there was a significant increase in the prevalence of type 2 diabetes from 1997 to 2011 [2]. Furthermore, the prevalence of diabetes rates in Saudi Arabia is predicted to increase over the next 20 years, which is similar to international trends [1, 3]. Between 2010 and 2030, the anticipated prevalence of diabetes in Saudi Arabia will be between 16.8 and 18.9% of the whole population [2, 3]. Within twenty years, it is therefore predicted that nearly one in four adults in Saudi Arabia will suffer from type 2 diabetes [4].

It is anticipated that the diabetes health crisis will cost the Government of Saudi Arabia a significant amount of money. According to Alhowaish, over the past 18 years, the rate of health care expenditure on diabetes care has increased by 500% to almost US$0.9 billion. For every $11 of Ministry of Health care dollars, $1 is spent on reducing diabetes [5]. It was estimated in 2010 that out-of-pocket expenditure for the management of diabetes, pre- and postdiagnosis, had increased tenfold per patient ($3686 versus $380) [5, 6]. This is because patients with type 2 diabetes require a continuous care programme involving follow-ups and check-ups; for example, HbA1c testing needs to be performed every 3 months, based on the US guidelines for managing type 2 diabetes [6]. Levinsky found that the early referral of patients with type 2 diabetes can reduce health care costs and can improve the quality of care [7]. Referral is defined as the action taken when a health worker or medical specialist at a certain level of the health system does not have sufficient resources at his or her disposal to treat that clinical condition; that is, they do not have either the skills or the drugs and equipment. As a result, assistance is sought at another facility where a professional with a similar or higher level of expertise can assume management of the client's condition [8].

The referral system in Saudi Arabia plays a major role in managing the flow of patients from primary to secondary and tertiary care (as the health care system structure is based on those three levels of care). According to the literature, the referral system has many problems (for instance, the lack of information in the referral letter). Regardless of its long history, no substantial improvements have been implemented to the referral system since its introduction. Therefore, the objective of this paper is to provide an assessment of the current referral system and identify how an efficient and effective referral system that is beneficial to the management for type 2 diabetes should be implemented in Saudi Arabia.

## 2. Method

**Figure 1:**
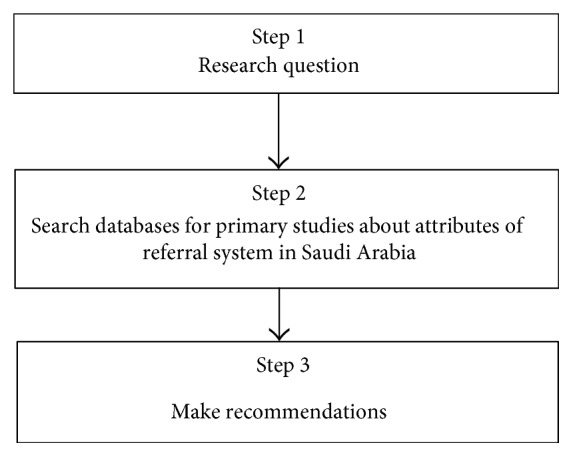
The three-step process for this study.

As shown in Figure 1, this study involves three major steps: the first step consists of formulating a research question, the second step is to conduct a systematic literature search within the Saudi context, and the third step consists of making recommendations for the referral system in Saudi Arabia.

### 2.1. Step 1: Research Question

A well-built clinical focus question should have a format that follows the PICO/PICOT concept. The PICO model is a tool that assists with organising and focusing questions into a searchable query and its elements help to identify search terms and concepts to use when searching the literature. In this paper, the concept has been modified to PIOT, as shown in Table 1.

### 2.2. Step 2: Literature Search for Primary Studies about the Attributes of the Saudi Referral System

The approach of a systematic review was selected as the research methodology for this project; however, the process was modified by having only one reviewer (the author), without a second reviewer. A systematic review was selected because, while literature on the referral system and type 2 diabetes in Saudi Arabia does exist, there are, however, no published systematic reviews of the referral system between primary and secondary health care for patients with type 2 diabetes and no recent comprehensive reviews since 2005. Therefore, the main purpose of this literature review was to determine the barriers to and the facilitators of an effective and efficient referral system. The systematic process of collecting and summarising the literature related to the selected topic is beneficial as it compiles the main findings of previous studies in the same field. The resulting review contains relevant information and research related to the topic of study and will help to identify gaps in the current research [9]. Therefore, in order to identify the solutions to the research question, a thorough review of the literature was carried out. The preliminary search was conducted on 1 March 2014, which retrieved 1880 articles.

### 2.3. Search Strategy

First, the three main indexing databases, PubMed, Embase, and CINAHIL, were searched (see Table 2). Second, the terms “diabetes mellitus, type 2” and “referral and consultation” were manually searched on the websites of three Saudi journals, *Saudi Medical Journal*, *Annals of Saudi Medicine*, and *Journal of Family and Community Medicine*. Third, Google Scholar and Google were searched using the same terms (“Saudi Arabia,” “diabetes mellitus, type 2,” and “referral and consultation”) for literature using similar Medical Subject Headings (MeSH) terms that might otherwise have been missed in the indexing databases. Fourth, in order to extend the search for specific relevant articles, backward and forward citation searching was used to identify key articles from the reference lists of articles. The search strategy was developed in consultation with an expert Liaison Librarian at Queensland University of Technology (Kelly Johnson). The databases and websites were identified and searched in March 2014.

### 2.4. Inclusion Criteria for Primary Studies

The inclusion criteria included Saudi Arabia, referral systems between primary and secondary health care, patients with type 2 diabetes, articles written in English, and, partly because the last comprehensive review article was published in 2005, a specified date range of 2004–2014. Further, this date range was used because, in 2004, the Saudi Ministry of Health began to focus more on chronic diseases and implemented changes to the current referral system, introducing a strategic plan to reduce the incidence of chronic diseases by providing special services.

### 2.5. Exclusion Criteria for Primary Studies

The criteria excluded articles that were not specifically related to the management of type 2 diabetes. This excluded articles relating to the pathophysiological features of type 2 diabetes that were not related to referral systems and management.

### 2.6. Screening of Primary Studies

Documents that were considered irrelevant as a result of their title and abstract were excluded. The full texts of the remaining publications were then prioritised and summarised, while the primary factors relating to referral systems were identified. Only papers that mentioned referral systems or related factors had data extracted from them.

### 2.7. Reporting of the Search Results

Transparent search strategies in systematic reviews assist with making an unbiased judgement of the findings [10]. Therefore, a flow chart was used that is intended to help authors meet the goals of wide-ranging systematic reviews and can lead to improved outcomes. The diagram outlines the flow of information through each section of a systematic review, with included and excluded records clearly identified by specified criteria [10].

## 3. Results

### 3.1. Primary Study Search

Initially, five main search terms were combined as follows: ((((“Diabetes Mellitus, Type 2”[MeSH]) AND “Referral and Consultation”[MeSH]) AND “Saudi Arabia”[MeSH]) AND “Primary Health Care”[MeSH]) AND “Secondary Care”[MeSH]. Three main databases were searched (PubMed, Embase, and CINAHIL), and there were zero results for the combination of these five terms. Further, when the two terms “primary health care” and “secondary care” were removed and the search was repeated, there were still no relevant results. Given that searching with three terms did not give any relevant results, two separate searches were conducted using “Saudi Arabia” as the main term and “referral OR type 2 diabetes” as the second term. After performing the two separate searches, it was possible to combine the findings and, as a result, 1277 relevant articles were retrieved from PubMed, Embase, and CINAHIL. The results are shown in Table 2.

Second, three significant Saudi journals (*Saudi Medical Journal*, *Annals of Saudi Medicine*, and *Journal of Family and Community Medicine*) were searched online to find any articles that might have been missed in the first search. These three journals had different searching methods; therefore, they were searched using only two terms, “referral” and “type 2 diabetes,” without the term “Saudi Arabia,” because these journals already relate mainly to Saudi Arabia. The results are shown in Table 3.

**Figure 2:**
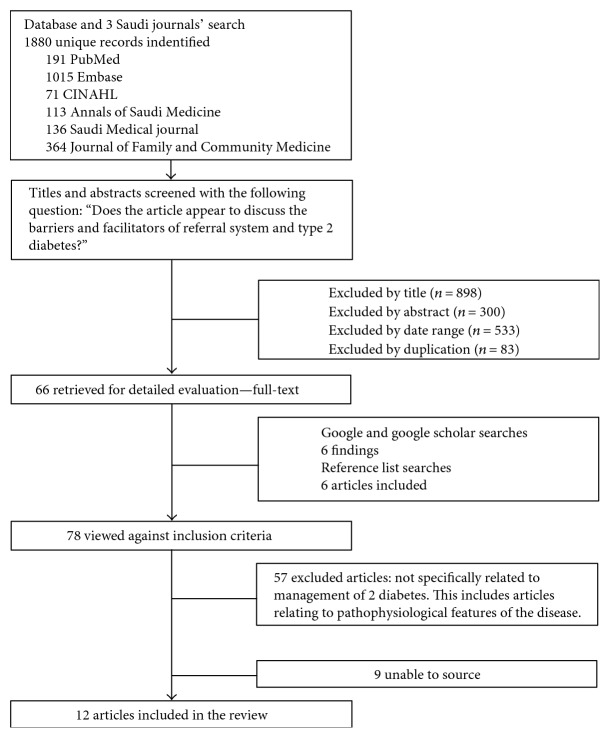
Selection process for inclusion of Saudi articles in the review.

Third, two different searches were conducted on Google and Google Scholar using “Saudi Arabia AND referral” and “Saudi Arabia AND type 2 diabetes”. There were a large number of search hits that were not related (over 6,000,000). Due to the large number involved, only the first two to four most relevant pages from the Google searches were used. In addition, many search hits were already included from previous searches and were excluded because of duplication. Therefore, only six possibly relevant additional articles were found, as shown in Table 4, and three of these were also later excluded. A summary of the selection process for the Saudi journal articles is shown in Figure 2.

Fourth, to extend the search for specific relevant articles, backward and forward citation searching was used to identify key articles from the reference lists of articles. First, the reference lists of the included articles were searched to find any articles that might be related to the referral system and type 2 diabetes in Saudi Arabia. Six articles were retrieved using this technique.

Overall, 77 articles were assessed against the inclusion criteria; however, only 12 articles met these criteria. Second, a forward citation search was conducted on these 12 articles using Google Scholar. Some articles had no citations at all and while the remaining articles with citations were reviewed, no new articles were found.

### 3.2. Overview of Primary Studies

Twelve articles were reviewed and included in this study, as shown in Table 5 [11–22]. All of the articles were published between 2004 and 2014. Eight were cross-sectional, three were retrospective studies, and one was a narrative review. The research described in the articles was conducted in seven different areas of Saudi Arabia: Holly Makkah (1), Riyadh (3), Tabuk (1), Gurayat (1), Qassim (2), Eastern province (3), and Sharurah (1). Therefore, the studies cover the north, south, east, west, and centre of Saudi Arabia. The dates of the studies were 2004 (2), 2005 (1), 2007 (4), 2009 (1), 2010 (2), 2012 (1), and 2013 (1).

Of the twelve articles, very few refer to the quality of the referral system in Saudi Arabia, although many studies focus on the quantity of referrals. The main goal of these studies is to decrease the quantity of referrals for cost effectiveness. The quality of referrals is not yet a world standard. The 12 studies establish that, while the referral rate is high, the quality of referral letters and feedback reports is inadequate and needs to be improved. There were also some factors relating to referrals that were not acknowledged at all in the articles, such as safety, timeliness, equity, quality, competency, and the degree to which the referral is patient-centred; all of which were introduced by the World Health Organization (WHO) and National Diabetes Education Program (NDEP) [8, 23].

Some studies recommended that a guideline should be available for physicians to improve the referral system. Therefore, there is an awareness of the problems with the referral system and a willingness to look at what should be done; however, no changes have been made to improve the system. The quality of those papers varies, but in general they were of poor quality.

The quality of patient referrals provides the linchpin for attempts to build a more integrated health care system. Most type 2 diabetes patients are older and comorbidities are common; therefore, high standard referrals should be timely and easily accessed. This study has used the existing literature to identify the referral issues that patients face daily in Saudi Arabia. We have located some of the main gaps in knowledge of the Saudi referral system, as applied to type 2 diabetes, indicating where there is a clear need for more research of higher quality on referrals for type 2 diabetes. The findings have a broader regional significance. These issues are also prominent in the Gulf countries, whose health systems are dealing with similar disease burdens, working within shared cultural values.

## 4. Limitations

Any systematic review can suffer from the potential weakness of relying on the quality of the included articles. The quality of the included Saudi studies was overall poor. Most of the studies included were cross-sectional, retrospective, and consisted of reviews. Cross-sectional studies can be biased because of the small sample size and, unfortunately, two of the included studies used cross-sectional methods and had small sample sizes [11, 19]. Therefore, one weakness in this paper is the lack of available information about important aspects of the Saudi referral system.

In addition, part of the problem, when studying the referral process in Saudi Arabia, is that the development of useful tools that could dramatically improve the referral process, such as the implementation of electronic health records [24], is still in its early stages, with very little supporting infrastructure. It is possible that this could change rapidly with the development of new types of electronic communication, but this issue should be the subject of further research.

## 5. Recommendations

The Ministry of Health in Saudi Arabia is putting a large number of resources towards funding better educated and better resourced health practitioners at all levels of health care and across multiple health care professions [25]. However, while this is a slow process, improved referral letters and processes for patients newly diagnosed with, and living with, chronic diseases such as type 2 diabetes could have a relatively high return value in terms of improved health outcomes.

While information exists on the rates of type 2 diabetes in Saudi Arabia and on the accuracy of early detection, there are no clear data on the rates of referral for early intervention compared to the rates of new diagnoses. More careful data collection on referrals, with a comparison to the number of people diagnosed, is required.

Further research on the quality of the health care system in Saudi Arabia is needed; however, there should be more consideration of the factors that relate directly to the patients' health outcomes. For example, the implementation of a safe, timely, effective, efficient, equitable, and patient-centred health system. The 12 Saudi studies were silent on some important global issues, such as equity of access. Therefore, further research is needed to focus more on the equity of access for people living in different areas of Saudi Arabia, for example, the marginalised rural population, the socioeconomically disadvantaged, and other minority groups.

## 6. Conclusion

This research tried to identify the gaps in the research on the efficiency of referral processes for patients with type 2 diabetes in Saudi Arabia. Overall, the majority of the Saudi studies included failed to mention the main referral factors for patients. Further research on the quality of the referral system, that takes into account at least some of the referral factors from the WHO guidelines, is required. The incidence of chronic disease is increasing in Saudi Arabia; therefore, more research on the services provided for people with chronic diseases is recommended.

## Figures and Tables

**Table 1:** Components of PIO-T for research question.

PIO-T model	
Population (P)	Patients with type 2 diabetes and other patients who need referral
Issues (I)	Factors of referral system between primary and secondary health care that affect patients with type 2 diabetes in Saudi Arabia
Outcome (O)	Better management, quality of life, reduced morbidity, reduced mortality, and any outcomes reported by the studies.
Type of study (T)	Cross-sectional, retrospective, and review.

**Table 2:** Database search.

Databases	PubMed	Embase	CINAHL
(“Saudi Arabia”[MeSH]) AND “Diabetes Mellitus, Type 2” [MeSH]	114 (76), Ex: 38 (date range)	1015 (853) Ex: 162 (DR)	20 (20) Ex: 0 (DR)
(“Referral and Consultation” [MeSH]) AND “Saudi Arabia” [MeSH]	77 (34) Ex: 43 (date range)	0	51 (39) Ex: 12 (DR)
((“Referral and Consultation” [MeSH]) AND “Saudi Arabia” [MeSH]) AND “Diabetes Mellitus, Type 2” [MeSH]	0	0	0
Total	1277		

**Table 3:** Saudi journal search.

Saudi journals	Annals of Saudi Medicine	Saudi Medical Journal	Journal of Family and Community Medicine
Referral	33 (33) Ex: 0 (DR)	55 (30) Ex: 25 (DR)	87 (49) Ex: 38 (DR)
Diabetes mellitus, type 2	80 (80) Ex: 0 (DR)	71 (57) Ex: 14 (DR)	277 (159) Ex: 118 (DR)
Total	603		

**Table 4:** Google and Google Scholar search results.

Searches	Google	Google Scholar
Referral and Saudi Arabia	1	2
Type 2 diabetes and Saudi Arabia	2	1
Total	6	

**Table 5:** A summary table of the included studies.

Author	Design	Sample	Result
Abdelwahid, Al-Shahrani, Elsaba, and Elmorshedi, 2010	Cross-sectional study	*N* = 452 (male = 183, female = 269)	This is a cross-sectional study conducted in 2010 in Sharurah by Abdelwahid, Al-Shahrani, Elsaba, and Elmorshedi. The main goal of this study was to assess the referral pattern and identify the appropriateness of the referral letter and consultant‘s feedback. Results indicated that there was lack of important information related to patients' medical history, physical examination, and investigation in the referral letters. Overall, there was a high percentage of referrals; however, the quality of referral letters and feedback reports was poor and needs to be improved.

Al Wadaani and Balaha, 2012	Retrospective study	*N* = 200	This is a retrospective study and conducted in 2012 in Al Hufuf city. The main objective of this study is to assess the appropriateness of physician responses in medical consultation reports and compare physician responses when using these reports from different levels of health care providers. Results indicated that almost half of referral is not safe for patients especially forms filled out by consultant and resident's physician. The form was semi structured and the authors recommended using structured letter [15]. This study may have information bias, because there were poor documented files as well as all variables available.

Alahmadi and Roland, 2005	A review	Not available	This is a review article that shows an overview of quality of primary care in Saudi Arabia. It was conducted by Al-Ahmadi and Roland in 2005. Overall, there was poor access for chronic disease management [12]. Also, the referral letters almost did not contain important medical information. Furthermore, they were handwritten and sometimes hard to read. There was low percentage of feedback sent from hospitals counting for only 22–39% of patients. As well, feedback reports lacked essential information including details of the advice given (100%), diagnoses (15%), or findings on investigations (21%).

Al-Alfi, Al-Saigul, Saleh, Surour, and Riyadh, 2004	Retrospective study	*N* = 4628	This retrospective study conducted in 2004 in Qassim region by Al-Alfi, Al-Saigul, Saleh, Surour, and Riyadh [20]. The main purpose of this study was to assess the quality of diabetic care of primary care in a rural town called Al Asyah. Results of this study indicated that there was high percentage of referrals, but the quality of referrals was inadequate. Therefore, it recommended that better coordination between primary and secondary care should be available for patients with type 2 diabetes as patients need services from both levels of care.

Al-Saigul, Abed-Elbast, Sourour, Ramzy, and Al-Alfi, 2007	Cross-sectional	*N* = 330	This cross-sectional study was conducted by Al-Alfi, Al-Saigul, Abed-Elbast, Sourour, and Ramzy in Buraidah in 2007 [22]. The study's main objective was to evaluate the quality of referral letters and feedback reports written according to the standards of Quality Assurance Manual of Ministry of Health from primary health care centers (PHCCs). Results indicated that there was lack of important medical information in referral letters. However, referral letters were better than feedback reports. The referral rate was very low for only 4%. The quality of referral letters and feedback was poor, and therefore, there should be improvements to the referral system.

Albattal, 2014	Cross-sectional	*N* = 51	This cross-sectional study was conducted in Riyadh in 2013 by Albattal [11]. It aims to identify the factors that contribute to inappropriate referrals and to suggest a better way to improve the quality of referrals. Therefore, 51 physicians were included in this study to give opinions about inappropriate referral. The sample size was very small in this type of study which increases a chance to be biased. Results indicated that one of the main factors to inappropriate referral was poor GP awareness about secondary care clinics. Another important factor was requesting referral by patient leading to inappropriate referral.

Al-Kaabba et al., 2010	Cross-sectional	*N* = 14138	This cross-sectional study conducted by Al-Kaabba, Abdalla, Saeed, AlZalabani, and Ahmad Mustafa in 2010 [18]. The main objective was to determine the referral pattern and characteristics of referred patients visiting military family medicine clinics in Tabuk, Saudi Arabia. Results indicated that there was a low percentage of feedback from consultants counting for only 13%. Also, females receive more referrals than male. There was no sample bias in this study because the sample size was big (14138). Overall, the quality of this study is good. Although this study was conducted in the military health sector, the results are applicable for general public health sector.

Ahmed, 2007	Cross-sectional	*N* = 430	This cross-sectional study was conducted in Gurayat in 2007 by Almoutaz [21]. The main objective of this study was to evaluate and compare the referral forms sent by primary care to diabetic clinics in secondary care with that of the American Diabetes Association. Results of this study indicated that the referral forms used were poor, as they did not reflect a clear picture of the referred patient. The author recommended that referral letters should be special and structured for patients with type 2 diabetes. The sample size was large which decrease the chance to be biased.

Al-Qahtani and Imtiaz, 2004	Retrospective	*N* = 138484	This retrospective study was conducted in 2004 by Al-Qahtani and Imtiaz [14]. The main objective was to analyse the pattern of referrals and evaluate the effect of clinical audit on the number and type of referrals from the primary care physicians to specialists. From the results, it seems that the authors focused more on the quantity of referrals but not the quality as that was mentioned by authors to decrease the cost. Also, the referral process was based on papers. Likewise, guidelines of referrals were not mentioned if disseminated to primary and secondary level; however, the authors mentioned that there was a decrease in the referral letter for two reasons. One of those reasons was increased awareness of physicians that irrelevant referrals will lead to increased workload.

Leena Baghdadi, 2007	Cross-sectional	49	This study was conducted by Leena and Rabab Baghdadi in the Holy Makkah city in 2007 [19]. The main purpose of this study is to evaluate the referral system from primary health care centers to hospitals and to improve the quality of the referral system and communication between the primary health care and the hospitals. This study has many biases. First of all, it is a cross-sectional study and this type of study should have a big sample. However, this study has only 49 participants. Secondly, the authors did not show the validity of the designed questionnaires. Finally, it has not addressed limitation. Overall, the quality of this paper is weak. However, results indicated that the referral system was poor and needed to be improved.

Khawaja et al., 2009	Retrospective-cross-sectional	*N* = 4616	This retrospective cross-sectional study was conducted in 2009 in Riyadh by Khawaja et al. [13]. The main objectives were to assess the referral rate of King Khalid University Hospital employees, from the employee's health clinic to specialty care, and to compare the rate of referral among both sexes of Saudi nationals versus expatriates. Results of this study indicated that most frequent reasons for referral were chronic problems (diabetes mellitus, hypertension, and bronchial asthma). Since, the main objective of this study was to decrease the rate of referrals, none of the 12 major referral factors were addressed.

Al-Ghamdi, Al-Turki, Al-Baghli, and El-Zubaier, 2007	Cross-sectional	*N* = 197681	This cross-sectional study was conducted in Eastern Province in 2007 by Al-Ghamdi, Al-Turki, Al-Baghli, and El-Zubaier [16]. The main objective of this study was to describe a community-based diabetes and hypertension screening campaign, the percentage of screened positive individuals, identified participation rate, and the factors affecting the participation. It includes urban and rural areas in Saudi Arabia. Results indicated that referral was higher from the rural areas than urban. Also, females have more successful referral than males. Primary care has the highest percentage of successful referral followed by other government hospitals. However, the MOH hospitals report very low successful referral.
